# Sampling Strategies for Three-Dimensional Spatial Community Structures in IBD Microbiota Research

**DOI:** 10.3389/fcimb.2017.00051

**Published:** 2017-02-24

**Authors:** Shaocun Zhang, Xiaocang Cao, He Huang

**Affiliations:** ^1^Department of Biochemical Engineering, School of Chemical Engineering and Technology, Tianjin UniversityTianjin, China; ^2^Key Laboratory of Systems Bioengineering, Ministry of Education, Tianjin UniversityTianjin, China; ^3^Collaborative Innovation Center of Chemical Science and EngineeringTianjin, China; ^4^Department of Gastroenterology and Hepatology, Tianjin Medical University General Hospital; Tianjin Medical UniversityTianjin, China

**Keywords:** sampling strategies, community structure, IBD microbiota research, feces, colonoscopic biopsy, mucus gel layer, oral cavity

## Abstract

Identifying intestinal microbiota is arguably an important task that is performed to determine the pathogenesis of inflammatory bowel diseases (IBD); thus, it is crucial to collect and analyze intestinally-associated microbiota. Analyzing a single niche to categorize individuals does not enable researchers to comprehensively study the spatial variations of the microbiota. Therefore, characterizing the spatial community structures of the inflammatory bowel disease microbiome is critical for advancing our understanding of the inflammatory landscape of IBD. However, at present there is no universally accepted consensus regarding the use of specific sampling strategies in different biogeographic locations. In this review, we discuss the spatial distribution when screening sample collections in IBD microbiota research. Here, we propose a novel model, a three-dimensional spatial community structure, which encompasses the x-, y-, and z-axis distributions; it can be used in some sampling sites, such as feces, colonoscopic biopsy, the mucus gel layer, and oral cavity. On the basis of this spatial model, this article also summarizes various sampling and processing strategies prior to and after DNA extraction and recommends guidelines for practical application in future research.

## Introduction

Inflammatory bowel diseases (IBDs), including Crohn's disease (CD) and ulcerative colitis (UC), are emerging as a part of a worldwide epidemic. CD was first diagnosed by Dr Burril B. Crohn (Crohn et al., 1932), in New York, in 1932, and UC was first described by White (1888), in Europe, in 1888. The former condition can cause inflammation in any digestive tracts, while the latter invariably affects the mucosa of the large intestine and rectum. Previous studies revealed that the prevalence of IBDs were greatly related to time (Molodecky et al., 2012), regions (Reinberg, 2015), age (Choi et al., 2015; Connelly et al., 2015), genes (Sharp et al., 2015; Wang and Achkar, 2015; Yang et al., 2015), stress (Gray et al., 2015), diet (Vagianos et al., 2016), etc., Some of these factors, including diet, were thought to be crucially connected to the genetic imbalance of the intestinal microbiota (Kosiewicz et al., 2011; Manichanh et al., 2012; Gevers et al., 2014; Kostic et al., 2014; Munyaka et al., 2016). Several studies have shown dysbiosis of the gut microbiome between patients with IBD and healthy individuals (Sokol et al., 2006; Andoh et al., 2012; Ottman et al., 2012). Owing to the decreasing cost and rapid development of next-generation sequencing (NGS) technologies (Zoetendal et al., 2008; Sheridan, 2014), the advancement of bioinformatics tools (Schloss et al., 2009; Caporaso et al., 2010; Glass et al., 2010), and the updating of online databases (DeSantis et al., 2006; Quast et al., 2013), 16S rRNA gene amplicon sequencing (Minamoto et al., 2015; Scher et al., 2015) and metagenomics analysis (Pérezcobas et al., 2014; Wang et al., 2015) have opened new frontiers to identify the variability of IBD microbiota research, which simultaneously characterizes multiple samples; it can also enable subsequent studies of microbial communities, both structurally, and functionally, while determining their interactions with the habitats they occupy.

Besides IBD, intestinal dysbiosis also plays a profound role in multiple chronic and metabolic diseases, including diabetes (Heintz-Buschart et al., 2016), obesity (Greenhill, 2015), irritable bowel syndrome (IBS) (Bennet et al., 2015), and so forth. Similar to IBD research; many studies conducted on the intestinal microbiota in relation to diabetes mellitus have predominantly used feces samples (Qin et al., 2012; Heintz-Buschart et al., 2016; Knip and Siljander, 2016). Additionally, in view of the connections between the periodontitis and diabetes mellitus, some studies have explored the diversity of subgingival microbiota between healthy controls and diabetics (Demmer et al., 2016). When investigating the relationship between intestinal microbiota and obesity, plenty of studies targeted the fecal microbiota for the reason that it is easily obtainable (Aguirre and Venema, 2015). Even though the small intestine is much more difficult to acquire than feces specimens, some researchers believed that sampling site should focus on the small intestinal microbiota, because it is where the calories are absorbed (Angelakis and Lagier, 2016). Moreover, a recent work showed that the obesity affected the subgingival microbial composition (Maciel et al., 2016). In IBS studies, the prevalently obtainable materials when sampling intestinal microbiota are feces and mucosal biopsies (Rangel et al., 2015; Parthasarathy et al., 2016). Accordingly, each disease has suitable sampling methods depending on pathophysiology and feasibility of the operation. Compared with other diseases, spatial ecological patterns are evident in common diseases of the colon, including the distribution of UC, and CD, which make the sampling sources diversified in IBD research (Lavelle et al., 2015). Meanwhile, understanding how the potentially complex pathogenesis of IBD occurs requires the integration of tools from spatial ecology with comprehensive sampling sources to define microbial dysbiosis in various niches (Lavelle et al., 2013).

The human body is composed of many niches. Biogeography studies the patterns of biological diversity in different niches, varying in both time and space (Fierer, 2008). The selection pressures of biology and the environment, elucidated by biogeography, are thought to be responsible for shaping the various habitats in the body (Lavelle et al., 2016). The community structure of microbiota across spatial niches might be disturbed to different degrees and in association with various disease states. Without cooperation among the other dimensions of microbial ecology, it may be difficult to investigate subjective signals from disturbances in a single niche (Jeffery et al., 2012; Lozupone et al., 2012). The International Human Microbiome Project (HMP)^1^, with its sum total funding of $115 million, has showcased the distinct variations of the human microbiota in different community structures (Group et al., 2009). Other studies of the human microbiome have also characterized the bacterial biogeography of different habitats (Costello et al., 2009; Grice et al., 2009; Zhou et al., 2013). Numerous research initiatives have shown interpersonal variation in human-associated microbiota in IBD (Lavelle et al., 2015, 2016). Likewise, intrapersonal variability has been discovered between different niches. Currently, the bacterial diversity in IBD research is determined by analyzing different community structures, and following the various aspects of feces (Kolho et al., 2015; Norman et al., 2015), colonoscopic biopsy samples (De Cruz et al., 2015; Rossen et al., 2015), and the mucus gel layer (MGL) (Johansson, 2014; Johansson et al., 2014). To obtain the MGL, researchers often use rectal swabs (Araújopérez et al., 2012), microbiological protected specimen brushes (PSBs) (Lavelle et al., 2013), and laser capture microdissection (LCM) (Lavelle et al., 2015). Recent research studies have indicated that oral microbiota will be used in clinical and diagnostic utilities (Yoshizawa et al., 2013; Said et al., 2014). Despite very promising prospects in the future, there is still no clear guidance identifying those methodologies that can be accurately used to systematically collect and process the samples. Some highly complex biological samples are often difficult to process, which can introduce much bias. These drawbacks can potentially influence the final result; yet, to comprehensively study the microbial diversity in IBDs, more information is indispensable in the design of spatial sampling strategies.

In this review, we focus on discussing the different sampling strategies used in IBD microbiota research from the perspective of three planes. Y-axis distribution includes the oral cavity and feces. X-axis gradients are distributed in intestinal biopsies, with sampling levels varying in the ileum, colon (ascending colon, transverse colon, and descending colon), rectum, and caecum. Z-axis distribution involves collecting luminal, mucosal, and mucous communities in a specific and regional manner, and it includes the feces, colonoscopy biopsy samples, and the MGL. Starting with a description of the y-axis distribution, we discuss the classic sampling sites—feces and the oral cavity. We then describe the x-axis distributions of colonoscopy biopsy. Ultimately, we will concentrate on the different sampling methods used for the MGLs, which are located on the z-axis. We herein provide an overview of the most crucial sampling strategies to help researchers make informed decisions.

## Sampling sites distributed along the Y-axis

### Feces

In the 1680s, Leeuwenhoek first described fecal bacteria using homemade microscopes (Egerton, 2006). With the rapidly evolving research on IBD in the nineteenth century, fecal flora was frequently used to represent intestinal microflora, as it was easily collected in patients. *Firmicutes* and *Bacteroidetes* phyla constitute the majority of dominant fecal microbiota using 16S rRNA amplicon sequencing, and with *Bacteroides* being the most abundant (Arumugam et al., 2011). Some work suggested that fecal bacterial communities could be divided into three enterotypes (*Bacteroides, Prevotella*, and *Ruminococcus*; Arumugam et al., 2011; Wu et al., 2011). Nowadays, fecal microbiota transplantation (FMT) has been widely used in the treatment of patients with IBD, which was found to be an effective therapy for some recipients (Kelly et al., 2015; Ince et al., 2016; Vermeire et al., 2016); thus, it was concluded that there should be some close connections between fecal microbiota and IBD. Probert et al. (2014) compared IBD patients and animal models of colitis with healthy individuals, and they found that the volatile organic compound (VOC) in feces held a potential role in identifying a novel diagnostic method for IBD. With a high sensitivity to inflammatory states, bacterial biomarkers in stool may therefore constitute a promising non-invasive source to diagnose IBD (Berry et al., 2015). In IBDs, the pH progressively increases along the duodenum to the terminal ileum; it decreases in the caecum, and then slowly rises from the colon to the rectum (Nugent et al., 2001). Such changes in colonic physiology are possibly reflected in the microbiota. Additionally, important factors such as diet (Lee et al., 2016), physical exercise (Queipoortuño et al., 2013), smoking habits (Biedermann et al., 2013), and antibiotic use (Pérezcobas et al., 2013) should exert subtle differences on fecal microbiota composition; of these, antibiotic use has a strong impact on one's initial microbiota composition (Macfarlane, 2014; Zhang et al., 2015b). Consequently, all of these issues shall be considered prior to sampling.

#### Sampling operating procedures

In view of the importance of the fecal sampling method, the study of the standard operating procedures (SOP) used to collect the fecal specimens has been, and still is, crucial for identifying pathogens. In the early stages, Moore (Moore and Holdeman, 1974) pointed out that some unique problems may arise with respect to the isolation and identification of intestinal bacteria in fecal flora studies, including collection, shipping, and isolation. Some experiments confirmed that the collection procedures and storage conditions did influence the diversity and integrity of the microbial flora (Cardona et al., 2012; Gorzelak et al., 2015; Boers et al., 2016; Nishimoto et al., 2016). It has been suggested that stool consistency is strongly associated with gut microbiota diversity (Vandeputte et al., 2016).

**Figure 1:**
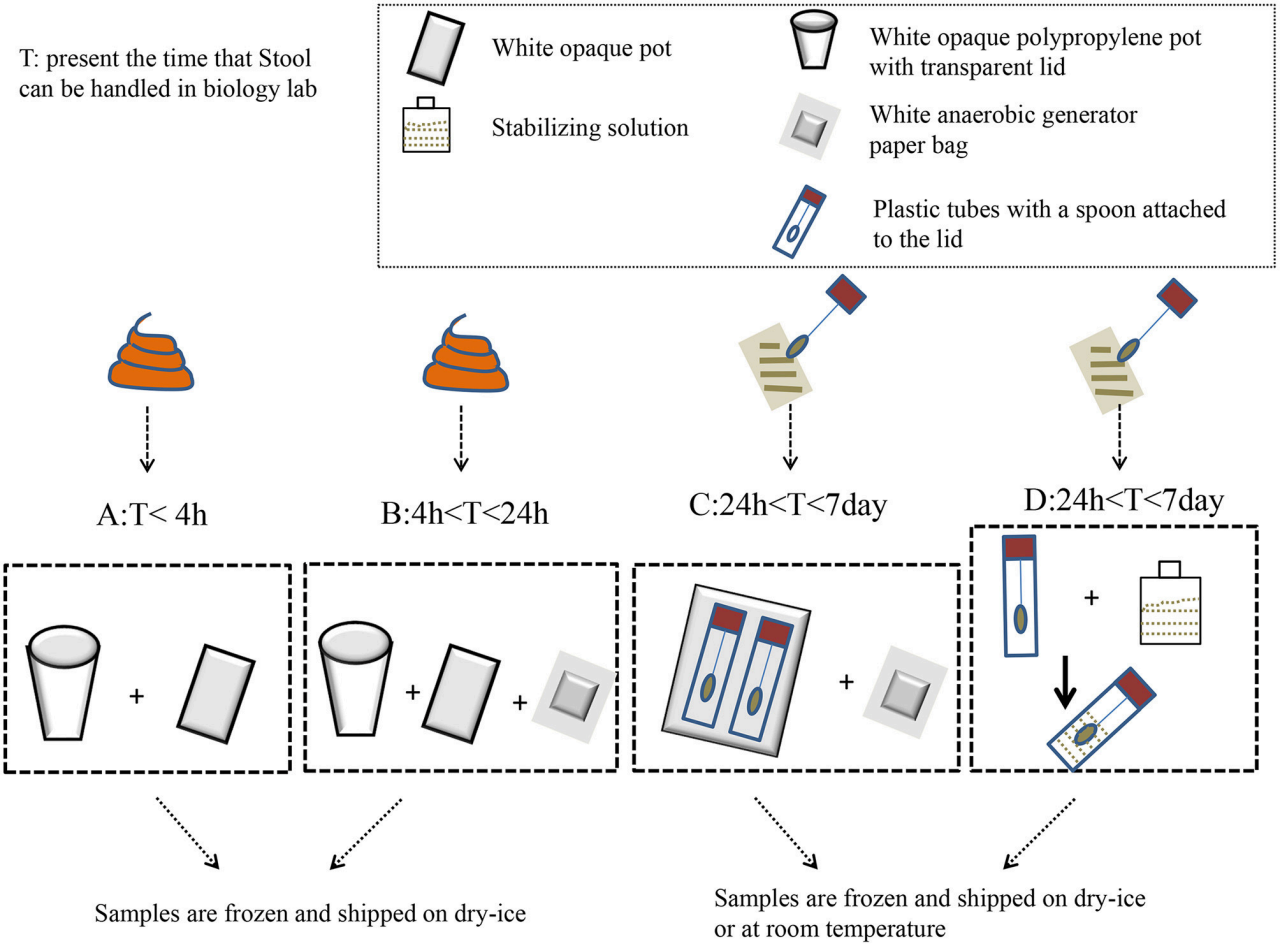
**The impact of methods that can be used to collect feces before laboratory handling**. When the fecal samples are transported to a biology lab within 4 h, they only need to be placed in a white opaque polypropylene pot with a transparent lid and a white opaque pot to hold the bag. Then, the samples are frozen and shipped on dry–ice to the lab **(A)**. When the samples can be brought to the laboratory within 4–24 h, tools should be used to add a white anaerobic generator paper bag on the basis of A to maintain a anaerobic atmosphere **(B)**. The plastic tubes, which have a spoon attached to their lids, are used to collect feces when the transit time is longer than 1 day. Fill up to two-thirds of the spoon with feces, and do not overfill. Then, the spoon and anaerobic generator paper bag are inserted in the opaque plastic bag **(C)**. Plastic tubes containing the stabilizing solution can keep the fecal DNA stable at room temperature for a few days (**D)**.

Swidsinski et al. (2008a,b) developed a new method using a punched-out freshstool cylinder; they demonstrated that the fecal flora were highly structured and spatially organized. The homogenization step in this procedure significantly reduced the intra-individual variation in the detected bacteria (Hsieh et al., 2016). Specifically, the results indicated that the relative abundance of *Firmicutes* to *Bacteroidetes* was significantly higher when snap-freezing fecal samples were compared with fresh samples (Bahl et al., 2012). Meanwhile, a study recommended that stool should be frozen within 15 min of being defecated, and it should be stored in a domestic, frost-free freezer for <3 days before DNA extraction (Carroll et al., 2012). During storage and processing, freeze–thaw cycles were detrimental to microbial cell integrity (Cardona et al., 2012). Conventionally, samples can be stored at −80°C in the long term until DNA extraction (for no longer than 6 months; Carroll et al., 2012). Based on the above, the ideal storing procedure might be as follows: homogenizing prior to sampling, sampling aliquot fresh stool to avoid subsampling; and then freezing at 80°C as soon as possible. If the laboratory has difficulty snap freezing, some researchers believe that RNAlater® (Life Technologies) might be selected to maintain DNA stabilization at +4°C, or even at room temperature, for several days without affecting the 16S rRNA repertoire (for specific treatments, see Figure 1). However, a new study suggested that RNAlater should be avoided due to its ability to degrade the yield of DNA and bacterial taxa (Gorzelak et al., 2015). Otherwise, a guanidine thiocyanate solution might ensure the high stability of fecal microbiota at room temperature (Nishimoto et al., 2016). Despite this, there are still no universally accepted standards in the field of feces sampling.

#### Sample extraction

According to the instructions and manual operation, 100 or 200 mg were the most frequently used dosages. One study showed that a 200 mg starting weight produced significantly higher DNA yields than 100 mg (Claassen et al., 2013); however, there was no similarity with respect to DNA purity. Conversely, Ariefdjohan (Ariefdjohan et al., 2010) tested 10–50 mg fecal samples and found that these weights, and not 100 mg or 200 mg, could result in maximum DNA yields. The phenol: chloroform-based DNA isolation method was illustrated to effectively obtain the requisite DNA yield (Mackenzie et al., 2015); however, this method is not suitable for clinical or large-scale studies. Owing to the bead-beating step, hot phenol with bead beating resulted in a proportional increase in *Firmicutes* (Wu et al., 2010; Mackenzie et al., 2015).

With respect to DNA extraction kits, those associated with the HMP view the MoBio PowerSoil® DNA Isolation Kit as the most effective microbial DNA extraction method. Moreover, some researchers involved in the International Human Microbiome Standards (IHMS; http://www.microbiome.standard.org/) prefer to use the QIAamp DNA Stool Mini Kit. Some researchers have conducted several studies on different extraction methods. As a result, the combination of mechanical cell disruption by repeated bead-beating (Yu and Morrison first described the repeated bead-beating and column purification method, Yu and Morrison, 2004) for 6 min, (Salonen et al., 2010) and with a 95°C heating step, showed greater bacterial diversity; it resulted in the significantly improved DNA extraction abundance of archaea and some bacteria, especially for bacteria in the phylum *Firmicutes*, including *Clostridium cluster IV* (Salonen et al., 2010; Thomas et al., 2015). However, bead-beating for long periods of time had a negative effect on DNA yield, and zirconium–silica beads were considered to be the best choice (Salonen et al., 2010). Due to the aromatic acids that exist in stool, some inhibition removal technology or substances were utilized to prevent interference—such as the inhibitEX tablets in the QIAamp DNA Stool Mini Kit (Thomas et al., 2015). Additionally, the size of the spin columns may also influence filter efficiency; for instance, sizes smaller than 0.45 μm would hold back some larger fragments (Thomas et al., 2015). Several studies have compared various DNA extraction kits and methods to assess the bacterial diversity in stool samples (Wu et al., 2010; Claassen et al., 2013; Kennedy et al., 2014; Mackenzie et al., 2015; see Table 1). It was found that finding a protocol to extract DNA without bias is a challenging task.

**Table 1:** **Overview of different processing methods or commercial DNA extraction kits that were compared in some studies to extract DNA from stool samples for further bioinformatics analysis**.

**序号**	**Kit/method**	**Sample store condition**	**Sample homogenization**	**Extra Lysis type**	**Inhibitor removal**	**Sequencing methods**	**DNA analysis**	**DNA yield**	**DNA purity**	**Bacterial diversity**
1 Wu et al., 2010	Hot phenol with bead beating+ QIAamp® DNA Stool Mini Kit	Immediately frozen (−80°C)	NO	Mechanical + Heat + Chemical + Enzymatic	YES	454 GS FLX and 454 Titanium	16V1–V2,V1–V3,V3–V5,V6–V9	B	–	The largest proportion of *Firmicutes*
	QIAamp® DNA Stool Mini Kit	Immediately frozen (−80°C)	NO	Mechanical + Heat + Chemical + Enzymatic	YES	454 GS FLX and 454 Titanium	16V1–V2,V1–V3,V3–V5,V6–V9	B	–	Similar to PowerSoil DNA Isolation Kit
	Stratec® PSP Spin Stool DNA Kit	PSP for 48 h, then frozen (−80°C)	NO	Mechanical + Heat + Enzymatic	YES	454 GS FLX and 454 Titanium	16V1–V2,V1–V3,V3–V5,V6–V9	A	–	With higher proportion of *Firmicutes*
	MoBio® PowerSoil DNA Isolation Kit	Immediately frozen (−80°C)	NO	Mechanical + Heat +	YES	454 GS FLX and 454 Titanium	16V1–V2,V1–V3,V3–V5,V6–V9	C	–	Similar to QIAamp DNA Stool Mini Kit
2 Mackenzie et al., 2015	Phenol: chloroform-based DNA isolation	Immediately frozen (−80°C)	YES	Mechanical	NO	Illumina MiSeq	16V4	A	B	With higher proportion of *Parabacteroides distasonis*
	QIAamp® DNA Stool Mini Kit	Immediately frozen (−80°C)	YES	Mechanical + Heat + Chemical + Enzymatic	YES	Illumina MiSeq	16V4	B	A	The largest proportion of *Bacteroidetes*
	MoBio® PowerSoil DNA Isolation Kit	Immediately frozen (−80°C)	YES	Mechanical	YES	Illumina MiSeq	16V4	A	B	With higher proportion of *Bifidobacterium adolescentis*
	ZR Fecal DNA Mini Prep TM Kit	Immediately frozen (−80°C)	YES	Mechanical	NO	Illumina MiSeq	16V4	B	C	The highest proportion of *Firmicutes*
	HMP Extraction Method	Pre-processed supernatant + 65°C 10 min, 95°C 10 min, then frozen at −80°C	YES	Mechanical + Heat	YES	Illumina MiSeq	16V4	C	B	The lowest proportion of *Firmicutes*, the highest proportions of *Cyanobacteria* and *Proteobacteria*
3 Kennedy et al., 2014	MoBio® PowerSoil DNA Isolation Kit	65°C 10 min, 95°C 10 min, then frozen at −80°C	YES	Mechanical	YES	Roche 454 Titanium	16V3–V5	B	–	With higher proportion of *Bacteroidaceae, Ruminococcaceae* and *Porphyromonadaceae*
	FastDNA® SPIN Kit for Soil	65°C 10 min, 95°C 10 min, then frozen at −80°C	YES	Mechanical	NO	Roche 454 Titanium	16V3–V5	A	–	With higher proportion of *Enterobacteriaceae, Lachnospiraceae, Clostridiaceae* and *Erysipelotrichaceae*

A–C stands for the performance rank: A (best performance) to C (worst performance)

#### Sample sequencing

Two methods are frequently used for taxonomic classification of organisms that are found in microbiomes: 16S rRNA gene amplicon sequencing and metagenomic sequencing. 16S rRNA gene amplicon sequencing is increasingly being used to provide information about the compositions and the relative abundance of microorganisms and classify microbial communities based on amplification of 16S rRNA gene, both taxonomically and phylogenetically (Clarridge, 2004). To analyze 16S rRNA gene sequences from microbial communities, QIIME, Mothur, and LotuS have been widely used to process data from high-throughput sequencing (Schloss et al., 2009; Kuczynski et al., 2011; Hildebrand et al., 2014). Additionally, PICRUSt (http://picrust.github.com/) has been developed to predict metabolic pathways based on 16S data and a reference genome database (Langille et al., 2013). Although this approach is unable to outperform metagenomic sequencing, it can predict and compare probable functions across a large amount of samples from different niches. Meanwhile, it can reproduce functional information that shows highly similar to the metagenomic sequencing in the HMP and other data sets (Anonymous, 2013). Compared with 16S rRNA gene amplicon sequencing, metagenomic approach is able to identify some of the distinctive functional attributes encoded in intestinal microbiota and comprehensively characterize metabolic capabilities of the microorganisms (Gill et al., 2006). Several tools have been developed to process the metagenomic data, such as MetaPhlAn (Segata et al., 2012), HUMAnN (Abubucker et al., 2012), and TruSPADES (Hildebrand et al., 2014). All approaches have merits and drawbacks. 16S rRNA gene sequencing is more cost-effective and less time consuming than metagenomic sequencing. However, metagenome approaches enable the analyses of all kingdoms as well as viral sequences. The 16S rRNA gene captures broader range of microbiome diversity, but with a lower resolution and sensitivity compared with metagenomic (Poretsky et al., 2014). Limitations withstanding, 16S rRNA is limited by the biases inherent to PCR amplification, which results from the lack of truly universal primers and different copy numbers of 16S rRNA gene (Vallescolomer et al., 2016). As for metagenomic sequencing, it could be less efficient at detecting rare species in a microbial community compared with 16S rRNA. Metagenomic sequencing also requires advanced bioinformatics skills to process and analyze the data (Shakya et al., 2013).

Theoretically, the best analysis method currently available is metagenomics; however, its associated costly budget is not suitable for clinic settings or large cohorts, and it faces some limitations with respect to environmental interactions. As a result, it was found that until recently, 16S rRNA gene amplicon sequencing is often used as an exploratory step before metagenomic research. With respect to the sequencing, the 16S rRNA database only includes bacteria and archaea; yet, the absence of viruses and eukaryotes misses many pathogenic factors, which may bias the analysis. The smallest units of operational taxonomic units (OTUs) are species, so the strains resulting in antibiotic resistance, as well as mobile elements cannot be identified (Thomas et al., 2015). Besides, *Bifidobacteriaceae* are not well represented in some 16S V1–V3 analyses (Jumpstart Consortium Human Microbiome Project Data Generation Working, 2012). According to some investigations, the optimal choice for the variable regions in the 16S rRNA approach were V1–V3 and V3–V5, as the choice of a V6–V9 primer did not appear to efficiently cover the V6–V9 regions (Wu et al., 2010; Jumpstart Consortium Human Microbiome Project Data Generation Working, 2012). Otherwise, the amount of chimera increased and amplified the polymerase chain reaction (PCR) bias (Schloss et al., 2011). To reduce the bias of the PCR methods, and to minimize the errors introduced during sequencing, some researchers developed a method known as Low-Error Amplicon Sequencing (LEA-Seq) (Faith et al., 2013), which has been applied to QIIME. Next, for high-throughput sequencing, both 454 GS FLX and 454 Titanium sequencing methods can be used, depending on convenience (Wu et al., 2010). With read lengths of currently up to 2 × 300 bp and low sequencing costs, Illumina's MiSeq (Solexa) is increasingly becoming one of the most potential sequencing platforms worldly used in IBD research (Quince et al., 2015; Chung et al., 2016). It gathers the integration of cluster generation, sequencing, and data analysis in a single instrument and can analyze data within 24 h (as few as 8 h; Liu et al., 2012). For sequencing technology, instead of pyrosequencing technology applied to 454 sequencer, MiSeq leverages sequencing by synthesis. Compared with 454 platforms, the MiSeq has a higher throughput per run and a lower error rate but a shorter reads (Liu et al., 2012; Loman et al., 2012). At the start of the IHMS project, the SOPs of fecal sample self-collection, conservation practice, and formulated sequencing standards are crucial for better understanding the fecal microbiome and for optimizing data comparisons in clinical settings.

### Oral cavity

While feces are frequently used in IBD research, there are certain limitations associated with outpatient distaste for handling these samples. Yet, researchers seek a simpler, more efficient, and more acceptable method. Oral samples are an important option. The oral cavity is a complex environment that includes the saliva, the tongue, teeth, tonsils, the buccal mucosa, and gingival sulci, which are colonized by a number of molecular and microbial analytes and bacteria (Human Microbiome Project, 2012). The microbiota in the oral cavity has a multitude of opportunities to reach the gut (Rochet et al., 2007). Pittock et al. (2001) reported oral lesion in nearly half of children that were newly diagnosed with CD. Similarly, one prospective study found that more than 30% of children with CD had involvement of the mouth (Harty et al., 2005). Another study noted a significant decrease in the overall diversity in the oral microbiota of pediatric CD patients (Docktor et al., 2012). Some bacteria in the oral cavity have recently been investigated for their association with IBD (Yoneda et al., 2016); these bacteria can be analyzed as microbial biomarkers for evaluating pathologies of the oral cavity, such as *Campylobacter concisus* (Ismail et al., 2012) and *Fusobacterium nucleatum* (Swidsinski et al., 2009). Thus, using oral microbial diagnostics is not a novel concept. Nowadays, scientists pursue a timely, accurate, cost-effective, and non-invasive diagnostic method to detect IBD. In view of these, further research on the oral microbiota in IBD might hold potential clinical and diagnostic utility in the future (Docktor et al., 2012). In this review, two frequently used sampling origins are primarily discussed: saliva and subgingival plaques.

#### Saliva

The average adult produces more than 1,000 mL of saliva per day, which always flows into the gastrointestinal tract. Thus, it can be stated that the salivary microbiota affects the development of gut microbiota in some respects. The composition of salivary microbiota was found to be different between CD patients, UC patients, and healthy controls (Said et al., 2014). Furthermore, when analyzing the composition of the tongue, buccal mucosa, saliva, and stool microbiota in colitis patients, the saliva microbiota exhibited the most alterations in terms of abundance (Rautava et al., 2015). The dominant genera, *Veillonella* and *Haemophilus* were recommended to largely contribute to dysbiosis of salivary microbiota in IBD patients (Said et al., 2014). At the species level, *C. concisus* (Ismail et al., 2012; Mahendran et al., 2013) and *Mycobacterium avium Paratuberculosis* (Bruno and Isabelle, 2015) have been investigated for its role in saliva dysbiosis of IBD patients.

For sample processing, DNA yield and quality, as well as 16S rRNA/DNA products and representations of the microbial community from oral wash samples, were investigated by six commonly used commercial DNA extraction kits, utilizing either mechanical bead-beating or enzymatic methods for cell lysis (Wu et al., 2014). Researchers discovered that mechanical bead-beating extraction kits produced less total DNA when compared with the enzymatic methods. On the other hand, microbial diversity showed no difference by either mechanical bead-beating or enzymatic extraction methods. As non-invasive and informative as saliva sampling is, but now there are currently no universally accepted techniques for sample collection. Prior to sampling the saliva, one must clean the oral cavity by rinsing it with water; this is imperative to avoid the presence of contaminants (Yoshizawa et al., 2013).

#### Subgingival plaques

As a human microbiome community, dental plaques were initially observed by Leeuwenhoek (Dobell, 1932) over 300 years ago. Using combinatorial labeling and spectral imaging fluorescent *in situ* hybridization (FISH) to differentiate up to 15 fluorescent probes, Welch and colleagues (Mark Welch et al., 2016) showed, for the first time, the informative value of the oral microbiota biogeography at the micron scale. The fantastic color images that they created showed that the oral cavity acted as a “coaggregation.” Similar to the role of canopies in hedgehog structures, *Corynebacterium* primarily gathered in subgingival plaques and supragingival dental plaques. Zhang et al. (2015a) first combined subgingival plaques and feces to analyze the microbiota perturbed in disease, and they partly normalized after treatment; at the same time, the researchers strongly confirmed the overlap in the abundance and function of species at different body sites. This will lead to potential ways to use the supragingival microbiota community for diagnosis and prognosis. Several recent studies have demonstrated connections between the composition of IBD and periodontitis (Kelsen et al., 2013; Elburki, 2015; Agossa et al., 2016). Meanwhile, additional studies have illustrated the associations between the composition of the subgingival microbiota and IBD (Brito et al., 2013; Kelsen et al., 2015). By analyzing inflamed subgingival sites, which depends on the checkerboard DNA–DNA hybridization technique, researchers found that the levels of *Prevotella melaninogenica, Staphylococcus aureus, Streptococcus anginosus*, and *Streptococcus mutans* are higher in CD patients than in controls. Furthermore, UC patients harbored a greater abundance of *Staphylococcus aureus* and *Peptostreptococcus anaerobius* than controls (Brito et al., 2013).

Thus, it is essential to study and collect subgingival plaques. To do so, place cotton balls in such a way that they can clean out residual supragingival plaques, prior to the collection of subgingival samples. Collect the subgingival plaque in a tube with buffer, using a sterile Gracey curette to gather the targeted teeth of the mesio-buccal surface. Then, firmly close the cap on the tube and shake the tube for 5 s to entirely homogenize the sample distribution in the buffer. Finally, place the sample on ice and send it to the biology lab within 4 h (McInnes and Cutting, 2010). The HMP method uses the MoBio PowerSoil® DNA Isolation Kit; other researchers have used the MasterPure DNA Extraction Kit (Moutsopoulos et al., 2015), the FastDNA spin Kit (Kuehbacher et al., 2008), the PSP Spin Stool DNA Plus Kit (Kelsen et al., 2015), and others. Optimal methods for DNA extraction are still under development.

## Sampling sites distributed along the X-axis

### Colonoscopy biopsy

Accordingly, luminal microbiota and mucosa-associated microbiota have been reported to be different in IBD (Lepage et al., 2005; Morgan et al., 2012; Gevers et al., 2014). Fecal microbiota might not adequately represent bacterial communities at the epithelial interface. Colonoscopy biopsy is the most common sampling technique used to assess microbial niches associated with the intestinal mucosa; it was shown to play a crucial role in diagnosis, and it can distinguish between disease types in IBD (Salvatori et al., 2012). Mucosal biopsies sample multiple amounts of the submucosa, epithelium, and MGL. The most comprehensive method to analyze the mucosa-associated microbiota may be proctocolectomy. In fact, Chiodini et al. (2013) were the first to examine the microbial populations of submucosal tissues using proctocolectomy during active disease; they also discussed the submucosal microbiota and biotypes within CD. Some other works also elected to use tissue sections of the terminal ileum and colon, obtained during surgery, for this process (Kleessen et al., 2002; Neut et al., 2002). As accurate as proctocolectomy is, this method cannot be applied to most of IBDs, except on rare occasions. Therefore, a more suitable method to obtain the tissue should be colonoscopy.

#### Sampling spatial distribution and processing

**Figure 2:**
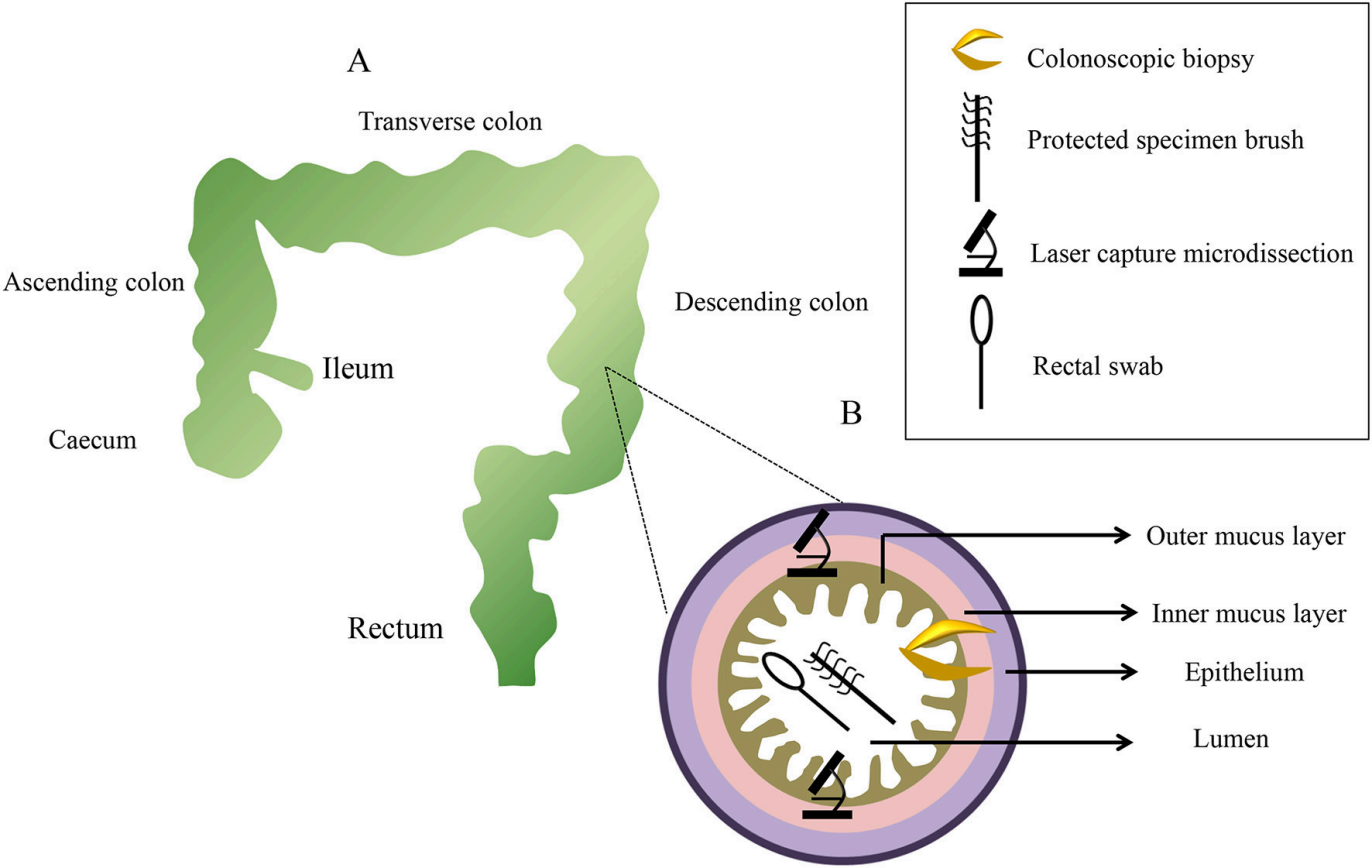
**A diagram of sampling sites distributed along the x-axis and z-axis with representative pictures from each sampling method**. Colonoscopic biopsy samples are collect from six levels: the ileum, ascending colon, transverse colon, descending colon, rectum, and caecum **(A)**. Samplings of the mucus gel layer occur at six sections using three methods **(B)**.

It has been said that diverse bacteria distribute heterogeneously along the small bowel to the colon (Eckburg et al., 2005). Biopsy specimens can be taken from different gut locations, such as the ileum, colon (ascending colon, transverse colon, and descending colon), rectum, and caecum. In addition, the intestinal tract contains a variety of distinct microbial communities along the ileum (around 155 cm from the anus), caecum (around 150 cm from the anus), ascending colon (around 142 cm from the anus), transverse colon (around 109 cm from the anus), descending colon (around 64 cm from the anus), and rectum (around 10 cm from the anus; Zhang et al., 2014), and the difference between longitudinal regions in the intestinal tract should be positioned to select the target regions for sampling (Figure 2A). Comparing the microbial diversity of samples obtained with sheathed forceps with those obtained with standard unsheathed forceps, biopsies from the specific sites were not contaminated with the work channel (Dave et al., 2011). Additionally, a novel biopsy technique (Brisbane Aseptic Biopsy Device) has been developed to prevent cross-contamination from intestinal luminal contents (Shanahan et al., 2016). To avoid the influence of biopsy specimen sizes of colonoscopic tissue, researchers quantified tissue cell numbers using primers of the β-globin gene to determine the total amount of mucosa-associated microbiota in the biopsy specimens (Wang et al., 2014b). Previous studies revealed that bowel preparation (PEG electrolyte solution) before endoscopy affected the composition and diversity of the tissue and stool samples (Harrell et al., 2012; Jalanka et al., 2015; Shobar et al., 2016). Dividing a single dose into two separate dosages may introduce fewer alterations to the intestinal microbiota, which is preferred in clinical practice (Jalanka et al., 2015). Still, bowel preparation may have little effect on the next sampling procedure, as it has a short-term effect on the composition of the intestinal microbiota (O'Brien et al., 2013). Once taken, some works suggested that biopsy samples were placed in a cryovial with a lid, immediately snap-frozen in liquid nitrogen, and then stored at −80°C until further analysis (van den Heuvel et al., 2015; Hedin et al., 2016; Munyaka et al., 2016). However, other mucosal biopsy specimens were harvested and then washed twice in 500 mL of phosphate buffered saline (PBS; pH 7–8) to ensure that there was no fecal contamination prior to being snap–frozen in liquid nitrogen (Shen et al., 2010; Sanapareddy et al., 2012; Budding et al., 2014; Berry et al., 2015). Considering the actual process, a protective solution can maintain the sample at −20°C for a few weeks, or at 4°C for 24 h (Zoetendal et al., 2006). Despite this, it is recommended that biopsy samples be processed as soon as possible to avoid the lysis of microbial cells.

#### Sample extraction and analysis

Quantities of bacterial cells in biopsy samples are 1% less than in feces samples (Lyra et al., 2012). DNA extraction procedures should be more carefully conducted in order to better represent the microbial community. A study that compared some DNA extraction methods, drew the conclusion that the bead-beating and column method, as well as high molecular weight methods, were likely to result in the increased production of DNA yield, which primarily included the *Firmicutes* bacteria (Ó Cuív et al., 2011). Nowadays, a large number of studies have preferred to use the QIAamp DNA Mini Kit for IBD biopsy DNA extraction (Hansen et al., 2013; Chen et al., 2014; Wang et al., 2014a; Lavelle et al., 2015). The positive effect of bead-beating on mechanical cell lysis has been discussed for fecal samples, which are sometimes also used in DNA isolation from biopsy samples (Chen et al., 2014). However, it appears that bead-beating may not require efficient microbial DNA extraction from biopsy specimens due to the fact that mechanical cell lysis of the biopsy specimens might increase the concentration of eukaryotic DNA, which may bias 16S rRNA gene sequencing analysis (Carbonero et al., 2011). A microbiome DNA enrichment method might potentially yield a higher fraction of microbial production, which methylated the human genomic DNA to selectively separate from microbial DNA (Yigit et al., 2016).

As for the spatial community structures (ileum, ascending colon, transverse colon, descending colon, and rectum) of human mucosal-associated intestinal microbiota, spatial variations of mucosa-associated microbiota have not provided feasible explanations to account for the observed longitudinal variations along the intestine, despite the previously observed spatial heterogeneity of mucosa microbiota (Aguirre de Carcer et al., 2011; Hong et al., 2011). Single-species abundance–distance dispersion (ADD) modeling results indicated that it was impossible to use conventional multivariate analysis methods to describe spatial heterogeneity and co-relationships across the multiple loci of microbial communities. The co-occurrence network analysis (Barberan et al., 2012) revealed a huge specialization among vertical and lateral gradients, and it addressed how interpersonal variation was a significant constituent of variance, particularly in light of the fact that the microbiota remains stable (Faust et al., 2012; Zhang et al., 2014). To reveal the longitudinal gradients in the microbiota along the x-axis distribution, studies may need to develop suitable statistical models and bioinformatics software.

## Sampling sites distributed along the Z-axis

### Mucus gel layer

**Figure 3:**
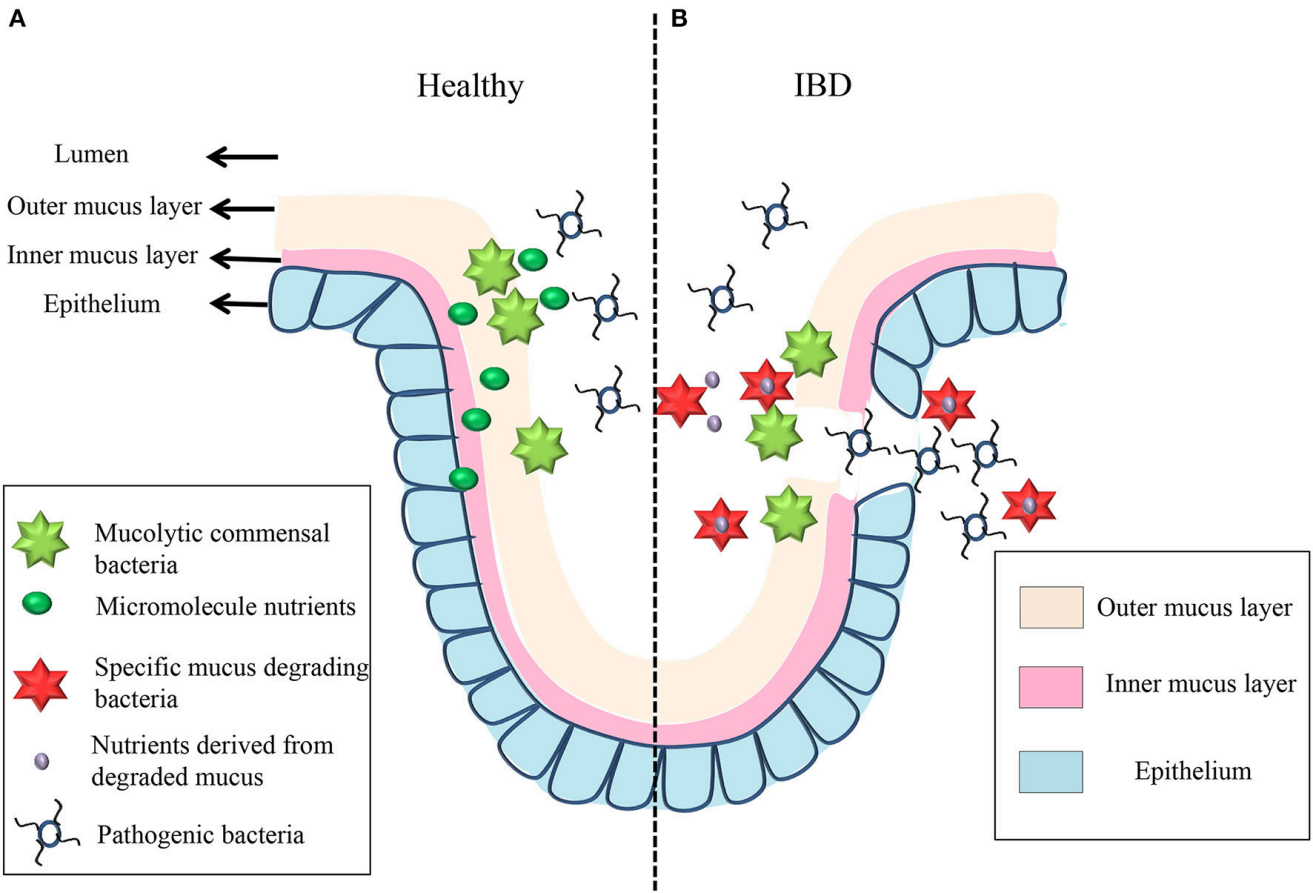
**The mechanism underlying mucin degradation in healthy individual and IBD patient**. In healthy individual, some commensal bacteria can bind to the outer mucus gel layer and act as a defensive barrier to resist pathogenic bacteria. At the same time, some short-chain fatty acids get through the mucus gel layers and epithelium to provide energy for mucus degradation, which is the first barrier between the lumen and the mucosa **(A)**. When inflammation occurs in IBD patient, some oligosaccharides derived from the degraded mucus offer energy to the mucus-degrading bacteria (like *Rumminococcus gnavus* and *Rumminococcus torques*); then, the invading bacteria change the mucus gel layer's structure, and pathogenic bacteria are now able to bind to and degrade the structure of the layers and invade the epithelium **(B)**.

Secreted by goblet cells that reside in intestinal crypts, the colonic MGL partially or entirely covers the epithelium and creates a boundary between the lumen and the host mucosa. Mucus is subsequently secreted and the layers fall off, generating a “district” that is carried into the fecal stream (Swidsinski et al., 2008b). The mucus is continuously secreted and can be divided into two layers: an outer, loosely adherent layer that can be removed by suction or gentle scraping; and an inner, firmly stratified layer that adheres to the epithelial cells (Atuma et al., 2001). In mouse models, the thickness of both MGL layers is appropriately estimated at 150 μm, with the outer layer measured at 100 μm and the inner layer at 50 μm (Johansson et al., 2008). The thickness of the human MGL is thought to be between 107 and 155 μm, depending on the loci (Pullan et al., 1994). Both layers are made up of MUC2-type mucin (Johansson et al., 2008). In healthy individuals, the inner layer is devoid of bacteria, while the outer layer serves as a habitat for the commensal microbiota (Hansson and Johansson, 2010; Johansson et al., 2011). The architecture of MGL exhibits a diverse range of polymers, including the mucus-binding protein (MUP), which offers numerous binding locations for both pathogenic and commensal bacteria (MacKenzie et al., 2009; Alemka et al., 2012). Some commensal bacteria are able to bind to and degrade the MUP, and they can be utilized as a barrier to pathogen binding. Mucin degradation of the MLG provides nutrients for some commensals, and it may initiate the initiation of pathogen invasion (Lennon et al., 2014b). As a result, the MGL plays a double role, providing a mutually beneficial environment for the host cells and resident microbiota, while serving as the first line of defense against pathogen bacteria translocating into the mucosa (see Figure 3). In IBD, bacteria are allowed to penetrate the inner MGL and reach the epithelium, triggering an inflammatory response; this suggests that the barriers of MUC2, with the absence of the MUC2 mucin polymer constituent, are disturbed, resulting in inflammatory responses (Schultsz et al., 1999; Swidsinski et al., 2007; Johansson et al., 2014).

On the basis of the aforementioned biological mechanism, identification of the mucus-degrading bacteria in the MGL is crucial. Conventionally, the MGL isolated from the precise fixation of intestinal biopsies or tissues, where dehydrating aldehyde fixatives are used, can result in loss and detachment of the mucus. Matsuo (Matsuo et al., 1997) demonstrated that using Carnoy's solution can preserve the integrity of surface mucus in paraffin sections of human colon specimens. Recent developments in overcoming this experimental limitation have achieved great success. Here, we describe three main sampling methods: rectal swab, the microbiologically protected specimen brush, and LCM. The vivid cross-sectional organization of each sampling method can be seen in Figure 2B.

#### Rectal swab

As a simple, standardized, non-invasive, and inexpensive method, rectal swab represents an important contribution when the patient does not wish to handle feces or undergo the discomfort and inconvenience of colonoscopy. A swab-sucked microbiota is reproducible, and the procedure can be performed by either the patient at home or by medical professionals in clinical settings; thus, this method may be suitable for clinical diagnostic purposes and clinical studies (Budding et al., 2014). Rectal swabs aim at collecting the colorectal mucus (Braun et al., 2009). Rectal swab specimens can be easily handled and stored immediately without perturbation of the microbiota. Swab specimens are obtained about 1–2 cm from the anal verge and collected by inserting a sterile cotton-tipped swab. This pioneering work suggested that swab sampling, without previous bowel preparation, harvested undisturbed microbiota (Budding et al., 2014). The swab was inserted into sterile PBS shaken for at least 2 min to ensure the sufficient release of microbiota, and the samples were then stored at −80°C until DNA isolation (Araújopérez et al., 2012); conversely, the samples could also be placed in tubes containing 500 mL of Reduced Transport Fluid buffer and maintained at room temperature for 2 h prior to storage at −20°C until DNA isolation (Syed and Loesche, 1972; Budding et al., 2014). For DNA isolation, the bead-beating step may have a negative effect on the estimated abundance of *Bacteroidetes* (Budding et al., 2014). DNA extraction kits can use the QIAamp DNA Mini Kit (Qiagen, Hilden, Germany) or Qiagen's DNeasy Blood and Tissue Kit (Araújopérez et al., 2012; Budding et al., 2014).

Previous work that has analyzed T-RFLP profiles and quantitative PCR (qPCR) has highlighted the differences in community diversity between samples obtained by biopsy or swab, and it was found that a higher abundance of *Lactobacillus* and *Eubacteria* were present in the swab specimens when compared with biopsies (Araújopérez et al., 2012). It was also previously demonstrated that *Staphylococcus aureus*, a dominant skin bacteria, could be used to assess the level of skin contamination between swabs and biopsies (Araújopérez et al., 2012). With respect to spatial organization, the fecal samples and swabs seemed to harbor more or less distinct diversity (Budding et al., 2014). One study revealed that the microbiota obtained by rectal biopsy and swab showed a greater similarity to one another than to feces (Glover et al., 2013). The diagnoses that are usually based on culture or NAAT on rectal swabs are widely utilized to distinguish between *Chlamydia* proctitis and CD (Hoentjen and Rubin, 2012). To prevent disturbances, from occurring, harvesting samples through a sheathed swab might lower the level of contamination by the skin and luminal microbiota in further studies.

#### Microbiological protected specimen brush

In recent research, a specimen brush was often applied to sample the human lung microbiota (Dickson et al., 2015; Schmidlin et al., 2015; Hogan et al., 2016; Sibila et al., 2016). Inspired by these investigations, Lavelle and colleagues (Lavelle et al., 2013) developed a novel sampling technique using the microbiological PSB for spatial microbial assessment; they targeted the superficial MGL from the luminal side, as it can fold over the light mucosa and avoid pools of fluid. Structurally, when compared with rectal swabs, this brush also targets an outer, colonized mucus layer that becomes separated from the epithelium via a dense layer of removable mucus. As a sterile, single–use sampling method, the brush is covered with a sheath, which consists of a distal plug at the tip to seal the brush when introducing and retracting the brush through the colonoscopy channel. After collecting the specimen, a sterile wire cutter is used to separate the tip of the wire and the plug, and the sample is then placed in a sterile, nuclease–free container until DNA extraction. The Qiagen DNA Mini Kit is frequently employed to extract DNA. The qPCR confirmed that the increased proportion of microbial DNA is sampled in the brush when compared with biopsy samples. Based on the 16S rRNA gene, the analysis of similarity analyses illustrated that there was a similar and highly significant difference between the PSB and biopsy samples, as well as between the Shannon Diversity Index values for reduced diversity in brush samples when compared to the biopsy samples (Lavelle et al., 2013).

#### Laser capture microdissection

Developed at the National Institutes of Health (Emmert-Buck et al., 1996), LCM is a systemic technique whereby individual DNA, RNA, and proteins can be sampled from the gut tissue by fixing targeted cells to an adhesive film with a laser beam; they are then observed under the microscope (Zhang et al., 2016b). LCM is a powerful method used to directly isolate pure sections from complex tissues with greater rapidity, specificity, and precision. This method does not require specific markers for identification, either prior to or after isolation, which is in contrast to rectal swabs and the microbiological PSB. To get at the MGL, researchers used LCM in healthy subjects undergoing a clinical routine colonoscopy, as well as in UC patients undergoing proctocolectomy for sampling (Lavelle et al., 2015), as based on the PALM MicroBeam system (Rowan et al., 2010a). Specifically, some researchers combined LCM and PCR to isolate and count the total amount of some mucosa-adherent bacteria, such as *Desulfovibrio* copies in the mucous gel of UC patients (Rowan et al., 2010a; Lennon et al., 2014a), as well as adherent–invasive *E. coli* from the macrophages of CD patients (Elliott et al., 2015). Given that *Mycobacterium avium subsp paratuberculosis* micro-organisms are few in number when present in CD patients, LCM was used to overcome this issue by accurately isolating subepithelial tissue, thus preventing contamination from the lumen (Ryan et al., 2002). Significant variations were observed between the colonic crypts and the central luminal compartment in mouse models, which used LCM to specifically profile the composition of the microbial communities in a discontinuous locus (Nava et al., 2011; Pedron et al., 2012). As a result, the study of colonic crypt mucus in UC patients, using LCM-harvested specimens, found that these patients had a lower abundance of crypt-associated bacteria than controls (Rowan et al., 2010b). Studies using LCM have placed standard and systemic histological sections of stained tissue under a microscope, and subsequently visualized the MGL of interest (Lennon et al., 2014a; Lavelle et al., 2015). Using a joystick to navigate around the image, researchers simply pushed a button to transfer the desired pure cells of the heterogeneous tissue to each slide to yield an average sample area of 175 mm^2^. Then, the LCM-harvested productions were catapulted onto an inverted opaque AdhesiveCap. As a targeted and specific quantified sampling method, LCM is suitable for research in precision medicine.

## Conclusion

As is well-known, suitable sampling strategies play an important role when studying the full landscape of intestinal microbiota. Here, this review highlighted the biogeographically stratified sampling strategies used in IBD, and it simultaneously proposed a novel three-dimensional spatial model of different community structures. Across these sampling sites, the non-invasive nature of fecal sampling can be implemented on a large scale as a screening or follow-up tool. However, feces are comprised of a mixture of products from all intestinal regions, which may not reflect the true nature of host–bacterial interactions in different biogeographic locations (Swidsinski et al., 2008b). Compared with fecal sampling, standard colonoscopy biopsy sample is sufficient to assess mucosal microbiota, which might affect mucosal and epithelial function to a greater degree than fecal sampling, as mucosal microbiota has a closer contact with immune cells and epithelial cells (Sartor, 2015). Furthermore, biopsy samples can be captured from specific regions ranging from the caecum to the rectum. These deep strengths notwithstanding, biopsy collection requires streamlining the logistics for sampling with nurses, physicians, and endoscopy technicians in advance to decrease the patients' time under sedation (Tong et al., 2014). The microbial profiles have indicated that at the early stage of disease, assessing rectal biopsy microbiota offered particular potential for convenient and early diagnosis of CD (Gevers et al., 2014). Particularly, in mouse studies, both tissue and feces sampling allowed targeted analyses of microbial under tractable and reproducible conditions. Fecal samplings could timely process feces to study the diversity of intestinal microbiota, varying in time (Zackular et al., 2013; Zhang et al., 2016a). Meanwhile, fecal pellets could also be collected from sacrificed mouse across different anatomical sites which often utilized caecal and colon contents (Bibiloni et al., 2005; Gaudier et al., 2005; Mishiro et al., 2013). Sometimes, the luminal content were flushed together by injecting PBS and then collected (Berry et al., 2012). The mucosa-associated microbiome is sampled by washing with PBS to remove the fecal contents then releasing epithelial cells (containing mucosal microbes) from the intestine tissue with mechanical means (Nagalingam et al., 2011; Tong et al., 2014). Specifically, LCM could specifically sample microbes that were located in the particular parts of mucosa (Nava et al., 2011). Evaluation of microbial community composition revealed striking differences between feces and tissues. The comparison between dextran sulfate sodium (DSS)-colitis mouse and controls showed that the 16S rDNA content (bacterial) was significantly decreased in feces but increased in mucosa, exhibiting the same trend as 18S rDNA (fungal; Qiu et al., 2015).

Coupled with the luminal microbiota, researchers have demonstrated that when using the MGL and entire mucosal biopsies, there is spatial variation in the intestinal microbiota, particularly among different community niches in UC patients (Lavelle et al., 2015). Moreover, human swab and colon biopsy samples have revealed that the mucosal diversity is prominent and enriched, particularly among the species from the phyla *Proteobacteria* and *Actinobacteria*, and when compared with the fecal microbiota (Albenberg et al., 2014). Zhou (Zhou et al., 2013) characterized the microbial variation between different community niches using a Dirichlet–Multinomial Distribution model, which concluded that feces and oral samples had the lowest interpersonal variability across the studied body sites studied in terms of community structure. To further illustrate this point, it has been reported that the numbers of bacteria in the *Clostridium coccoides* group remained stable in both feces and saliva over time (Singhal et al., 2011). Stearns et al. (2011) sampled species across the human digestive tract, including from feces, the stomach, colon, duodenum, and oral cavity, and illustrated that the oral cavity harbored the greatest phylogenetic diversity. Predictably, the oral microbiota holds great potential with respect to clinical and diagnostic utility.

Specific to mucosal biopsies and the MGL, there should be heterogeneity in the mucosal species that exist along cross-sectional and longitudinal axes of the bowel within specific individuals. However, due to the masking of a high level of individual variation, significant differences across longitudinal variations were not discovered by analysis of variance (ANOVA) (Zhang et al., 2014). Employing a multidisciplinary approach (such as by investigating ecological relationships and performing co-occurrence network analysis) may lift this mask of spatial variation to uncover the truth in prospective studies (Zhang et al., 2014). Specific to our study, we are devoted to developing statistical models to show the informative value of microbial biogeography in IBD research.

Traditional protocols are currently limited by the present difficulties associated with comprehensively evaluating the microbiota in IBD research. Such difficulties include fastidious experimental requirements and sampling errors. Therefore, it is critical that risk-free, standardized, simpler, and inexpensive sampling strategies be formulated in the future. To study potential contributions of the microbiota in IBD research, we should standardize the SOPs and reach a consensus that better facilitates our understanding of these methods in subsequent studies. Moreover, data should be exchanged and further studies should be designed in which we evaluate the microbiota within those individuals at the early stages of IBD. To construct a full picture of the microbial diversity in IBD research, synergistic profiles, combined with a co-culture consortium that can study bacteria, will be necessary. Comprehensively, it should be stated that a mutually beneficial cooperative effort can be achieved, but only if data on these methods are shared all over the world.

## Author contributions

SZ wrote the paper; XC and HH performed the collected the data. All authors listed, have made substantial, direct and intellectual contribution to the work, and approved it for publication.

### Conflict of interest statement

The authors declare that the research was conducted in the absence of any commercial or financial relationships that could be construed as a potential conflict of interest. The reviewer VJ and handling Editor declared their shared affiliation and the handling Editor states that the process nevertheless met the standards of a fair and objective review.

## Footnotes

^1^International Human Microbiome Standards (IHMS) project http://www.microbiome-standards.org/ [Online]. [Accessed].

## Abbreviations

IBD: Inflammatory bowel disease
CD: Crohn's disease
UC: Ulcerative colitis
NGS: Next-generation sequencing technologiesl
HMP: International Human Microbiome Project
IBS: Irritable bowel syndrome
FMT: Fecal microbiota transplantation
VOC: Volatile organic compound
SOP: Standard operating procedures
IHMS: International Human Microbiome Standards
OUT: Operational taxonomic units
PBS: Phosphate buffered saline
ADD: Abundance–distance dispersion
MGL: Mucus gel layer
MUP: Mucus-binding protein
PCR: Polymerase chain reaction
PSB: Protected specimen brush
LCM: Laser capture microdissection
ANOVA: Analysis of variance
DSS: Dextran sulfate sodium.
